# Social support criteria in vascularized composite allotransplantation versus solid organ transplantation: Should the same ethical considerations apply?

**DOI:** 10.3389/fpsyg.2022.1055503

**Published:** 2022-11-22

**Authors:** Laura L. Kimberly, Ogechukwu C. Onuh, Erika Thys, Eduardo D. Rodriguez

**Affiliations:** ^1^Hansjörg Wyss Department of Plastic Surgery, NYU Grossman School of Medicine, New York, NY, United States; ^2^Division of Medical Ethics, Department of Population Health, NYU Grossman School of Medicine, New York, NY, United States; ^3^University of Nevada, Reno School of Medicine, Reno, NV, United States

**Keywords:** social support, vascularized composite allotransplantation, ethics, equity, psychosocial

## Abstract

The field of vascularized composite allotransplantation (VCA) is evolving, with some procedures poised to transition from highly experimental research toward standard of care. At present, the use of social support as an eligibility criterion for VCA candidacy is at the discretion of individual VCA programs, allowing VCA teams to consider the unique needs of each potential candidate. Yet this flexibility also creates potential for bias during the evaluation process which may disproportionately impact members of certain communities where social configurations may not resemble the model considered “optimal.” We examine the extent to which ethical considerations for social support in solid organ transplantation (SOT) may be applied to or adapted for VCA, and the ethically meaningful ways in which VCA procedures differ from SOT. We conclude that VCA programs must retain some flexibility in determining criteria for candidacy at present; however, considerations of equity will become more pressing as VCA procedures evolve toward standard of care, and further empirical evidence will be needed to demonstrate the association between social support and post-operative success. The field of VCA has an opportunity to proactively address considerations of equity and justice and incorporate fair, inclusive practices into this innovative area of transplantation.

## Introduction

The field of vascularized composite allotransplantation (VCA) has evolved over the last two decades and now includes over 60 active hand, face, uterine and penile transplant programs (Cherikh et al., 2019). Much like in solid organ transplantation (SOT), the use of social support criteria for eligibility is at the discretion of individual VCA programs, providing ample leeway for VCA teams to consider the unique needs of each potential VCA candidate (Jowsey-Gregoire and Kumnig, 2016). Yet, this flexibility also creates the potential for bias to enter the transplant process at multiple touch points, including referral, evaluation, and listing (Ladin et al., 2019a; Mohottige et al., 2021; Reese et al., 2021; Park et al., 2022). Moreover, the very concept of social support in transplant is predicated in part on the presumption that an “optimal” social configuration exists that best positions transplant recipients to fare well (Maldonado, 2019). By extension, communities where social configurations and norms do not resemble the “typical” or “ideal” model may be disproportionately affected by both implicit and explicit biases, thereby exacerbating inequities in access (Maldonado, 2019; Ladin et al., 2019a).

Concerns about the ethics of social support criteria have been raised and debated in the SOT literature (Batra and Rubman, 2019; Berry et al., 2019; Beverley and Reischer, 2019; Fuller, 2019; Goldberg and Foster, 2019; Kelly-Hedrick and Henderson, 2019; Maldonado, 2019; McCauley and Fox, 2019; Parent, 2019; Priest, 2019; Wall, 2019; Ladin et al., 2019a; Mohottige et al., 2021; Reese et al., 2021). In this analysis, we explore the extent to which ethical considerations for social support in SOT may be applied to or adapted for VCA. VCA procedures differ meaningfully from most SOT in a number of ways that are ethically significant. The goal of VCA is to enhance rather than to extend life, and thus competing ethical principles ought to be balanced accordingly. Furthermore, VCA is still generally considered experimental and conducted as research, with implications for weighting ethical priorities that favor greater discretion for individual programs to ensure procedures are safe and effective. And finally, VCA types vary greatly from one another (with a higher degree of variation within each type), and recipients’ rehabilitative trajectories differ extensively, again necessitating more nuanced approaches to standards for eligibility criteria.

We consider how the ethical principles of utility and equity should be applied in VCA, and the tensions that arise when they are in conflict. We address the harms associated with bias and discrimination and review several alternatives for providing social support. We then conclude that a one-size-fits-all approach to social support as an eligibility criterion in VCA is unlikely to meet the varying needs of each type of VCA at present. Some discretion must be retained, particularly for lower-volume procedures such as face and penile transplants. That said, the field of VCA has an opportunity to proactively address considerations of equity and justice and can look to SOT for guidance on incorporating inclusive practices into this innovative area of transplantation.

## Balancing utility and equity in organ transplantation

The organ transplant system in the United States is guided by the distinct and sometimes competing principles of utility and equity (National Academies of Science, Engineering, and Medicine et al., 2022). Utility seeks to maximize the good that can be derived from available resources, in this case organs available for transplantation (Ethical Principles in the Allocation of Human Organs, 2015; National Academies of Science, Engineering, and Medicine et al., 2022). Given the perennial shortage of organs for transplant, distribution of this scarce resource must take into account where and for whom an organ will confer the most benefit (Clarke, 1995; National Academies of Science, Engineering, and Medicine et al., 2022). The principle of utility informs allocation policy to ensure that organs go to those who will benefit most, balancing need and likelihood of a successful outcome defined by numerous clinical endpoints including overall graft function, graft survival and mortality (Ethical Principles in the Allocation of Human Organs, 2015). At the same time, the transplant system weights these considerations of utility alongside moral obligations to promote equitable distribution of scarce resources and fair access to transplant (Ethical Principles in the Allocation of Human Organs, 2015). Criteria for transplant candidacy and organ allocation reflect the intermingling between considerations of utility and equity, including the use of social support criteria to determine eligibility for transplant.

## Social support and the problem of construct validity

Social support first gained attention in the literature as an important element in the relationship between stress and health outcomes (Cobb, 1976). Scholars have addressed social support from a multiplicity of theoretic vantage points, struggling to agree on a consistent definition. Social support can be defined variously as information, as a resource or resources, as availability of helping relationships, and as transactional resource provision, for example. Definitional confusion has resulted in heterogeneity in how the construct is conceptualized, operationalized and measured in the context of health and wellbeing (House, 1981; Chiaburu and Harrison, 2008; Ng and Sorensen, 2008). While existing empirical evidence suggests a link between social support and health outcomes (Cohen and Leonard Syme, 1985; Berkman and Glass, 2000; DiMatteo, 2004; Roth et al., 2005), the relationship between social support, health and wellbeing is not clear, due in part to aforementioned conceptual ambiguity (Kossek et al., 2001; Kim et al., 2005; Roth et al., 2005). Unsurprisingly, research in this area has focused on a variety of outcomes including behavioral, attitudinal, cognitive and/or emotional measures (Cohen and Leonard Syme, 1985; DiMatteo, 2004; Kim et al., 2005; Uchino, 2006, 2009). In the context of organ transplantation, lack of clarity about how to define, operationalize, and measure social support makes it difficult to assess the impact of social support on transplant outcomes, leaving room for bias and discrimination in assessment of eligibility for transplant procedures. This is the case not only in SOT, but also in evaluation for VCA candidacy.

## Social support in SOT: Empirical evidence

Approximately 30,000–40,000 organ transplant procedures are performed annually from both living and deceased donors, only 3–20 of which are VCA (Transplant Trends, 2022). To date, no guidelines explicitly define optimal social support in the setting of transplant surgery (Ladin et al., 2019a). In Ladin et al. (2019b) reported different definitions of social support among psychosocial clinicians ranging from informational, emotional, instrumental, motivational, financial, and importance of the patient to others (Ladin et al., 2019b). A study published as recently as 2021 determined statistical significance between social support and medication adherence following SOT but did not assess whether the correlation was due to emotional support or the direct management of medications by loved ones (Huang et al., 2021).

In an article published in the American Journal of Bioethics (AJOB), Berry et al. examined the ethics of social support as a criterion for access to SOT, grounding their analyses in existing empirical evidence addressing the association between social support and transplant outcomes (Berry et al., 2019). They found the empirical evidence linking social support to transplant outcomes insufficient and concluded, therefore, that lack of social support alone should not prevent an individual from accessing life-saving SOT. Furthermore, they suggested the criterion, as presently deployed, inappropriately favors utility and undermines important equity considerations. Specifically, formalized social criteria risk exacerbating the societal disadvantages inherent in marginalized communities, particularly those with reduced health care access and benefits, absent or non-traditional sources of social support, and lower income, thereby not being able to afford aspects of post-operative care such as medications, a live-in caregiver, or transportation to follow up appointments.

However, social support is inextricably linked to patient selection in SOT as surgical outcomes are dependent on the post-operative care period (Ladin et al., 2019a). Despite the importance of post-operative support in SOT, there are no guidelines formally in place in the literature to assist clinicians and transplant programs in establishing standardized approaches to incorporating assessment of social support in the evaluation process for transplant candidacy.

## Social support in VCA: Empirical evidence

Current empirical evidence on the impact and validity of social support criteria for VCA is scarce. Although a 2013 study documented statistical significance for patients with adequate documentation of social support system and transplantation failure rate of primarily hand and face, the only definition of social support provided was “suitable resources to sustain medication, adjunctive therapies, and follow-up” (Zhu et al., 2014). In other published VCA research mentioning social support, the criterion is described variably as “strong support from family and community”(Benedict and Magill, 2018), family members who may need to prepare for the media attention that often comes with VCA cases (Kumnig and Jowsey-Gregoire, 2016) or caregivers to aid in tasks of daily living, caring for the patient’s children, and providing financial support during recovery (Kumnig and Jowsey-Gregoire, 2016). These heterogeneous considerations further demonstrate the subjective nature of social support as a requirement for VCA eligibility. Finally, the presence of caregivers during the consultation and operative journey as well as mental health screening are discussed in the current VCA literature as important components in establishing psychosocial support (Kumnig and Jowsey-Gregoire, 2016).

## Discussion

The ethics discourse sparked by Berry et al.’s target article and accompanying commentaries offers a lens for comparative examination in the context of VCA (Batra and Rubman, 2019; Berry et al., 2019; Beverley and Reischer, 2019; Fuller, 2019; Goldberg and Foster, 2019; Kelly-Hedrick and Henderson, 2019; McCauley and Fox, 2019; Parent, 2019; Priest, 2019; Sharma and Johnson, 2019; Wall, 2019). To adapt ethical arguments about the appropriate use of social support in SOT for application in VCA, we examine ethically meaningful differences between SOT and VCA and ways in which these differences will likely shift over time.

Given the recent evolution of the field of VCA and the small number of procedures that have been performed to date relative to SOT, evidence in the form of long-term outcomes data to support the appropriate role of social support in VCA patient selection is even more scant than in SOT, particularly for lower-volume procedures such as face and penile transplants. And yet, variation in frequency and volume of procedures performed across VCA types means that some higher volume VCAs (hand and uterus) are approaching the transition toward standard of care. For those further along this trajectory, it will become increasingly important to standardize eligibility criteria and shift the focus from the ethical principle of utility toward equity to optimize patient care (Kimberly et al., 2019).

### Ethically relevant differences between SOT and VCA

How do these considerations about the appropriate use of social support criteria in SOT translate to the VCA space? To answer this question, we address characteristics of VCA that differentiate this innovative area of transplantation from SOT.

#### Life saving versus life enhancing

Unlike in SOT which in most cases is considered life-saving, enhancing quality of life is the primary goal in VCA procedures. The ethical considerations of VCA necessitate an alternate lens than SOT, as the risk to benefit ratio for VCA differs and thus requires a different weighting of priorities (Kumnig and Jowsey-Gregoire, 2016). While some benefits of VCA involve significant improvements in function and reduction of pain, other benefits are more psychosocial in nature and include the possibility of social reintegration and considerations for a recipients’ sense of identity. This is particularly the case in the context of face transplant, pre-and post-operative body image, and quality of life (Kumnig et al., 2014). Such concerns warrant prioritizing and considering the potential psychosocial harms that come with disfigurement. It might be argued that, for a procedure considered life enhancing but not life-saving, social support could be perceived as carrying more importance as an additional safeguard to protect against potential risk. In the case of a life-saving procedure such as SOT, where the alternative to transplant is death, limited social support might not weigh as heavily against the risk of not proceeding with transplantation. Thus, assurance of robust social support may have a greater role to play at present in patient selection for VCA than in SOT.

#### Balancing utility and equity in experimental research versus innovative therapy

The clinical research context generates ethical considerations that are distinct from considerations encountered in clinical practice. With respect to patient selection, while research efforts tend to lean more toward utility with the goal of selecting the “optimal” patient to ensure the best possible outcomes and establish proof of concept (Maldonado, 2019), the pendulum will shift toward ensuring fair access once a procedure is well established. Concerns around equity in SOT are at the forefront at present, hence the calls to interrogate the definition and operationalization of social support and its relation to transplant outcomes to ensure that social support criteria are not discriminatory and are grounded in solid evidence (Zhu et al., 2014; Kumnig and Jowsey-Gregoire, 2016; Benedict and Magill, 2018; Ladin et al., 2019a). While some forms of VCA, particularly hand and uterine transplant, are poised to transition toward standard of care, others are still considered highly experimental meaning that ethical considerations of utility still guide approaches to patient selection.

#### Variation within VCA

VCA types vary greatly by total volume of procedures performed and by rate of performance over time. Moreover, as previously noted, VCA types differ in their status on the developmental trajectory from highly experimental research to innovative therapy approaching standard of care (Diep et al., 2021; Jones et al., 2021; Lake et al., 2022; Wells et al., 2022).

##### Upper extremity

To date, approximately 148 hand transplants have been performed worldwide (Wells et al., 2022). As a relatively high-volume form of VCA, hand transplant is poised to shift toward standard of care. This procedure has the potential to scale up, and the complexity of the procedure itself is fairly consistent from one case to another, thereby enabling programs to develop expertise.

##### Face

As compared to hand transplant, face transplant is a resource-intensive, low-volume procedure and is likely to remain so for the foreseeable future. Only 48 face transplants have been documented worldwide (Diep et al., 2021). Each case presents a range of unique technical challenges, and each procedure must be carefully tailored to the specific recipient’s needs and anatomical characteristics (including natal characteristics and changes to anatomy as a result of injury or disease) and the anatomy of the deceased donor.

##### Genitourinary

Uterine – Compared to other forms of VCA, the volume of uterine transplants (UTx) performed annually has increased steeply in a relatively short period of time. To date, data for over 70 UTx have been published (Jones et al., 2021). This is due in part to the nature of UTx, which arguably may be considered more akin to solid organ transplants (Johannesson et al., 2014). Procedures are less variable, allowing for a more rapid development of experience within a UTx program and thus capacity to scale up. The donor-recipient matching process differs from other forms of VCA, without the aesthetic considerations of externally visible hand, face, and penile grafts. UTx is distinct from all other forms of SOT and VCA in that the grafts are intended to be temporary, with removal *via* a second surgery following successful achievement of pregnancy and live birth. Among the various types of VCA, UTx has approached standard of care most rapidly and, in fact, Baylor University in the United States now offers UTx as clinical care outside of a research protocol. However, the procedure is only available to individuals who can pay out of pocket, as commercial insurance has not yet approved reimbursement for all costs associated with UTx.

Penile – At present, only four penile transplants have been performed worldwide and detailed in the literature (Lake et al., 2022). The procedure is still considered highly experimental, and it is unclear whether it may eventually become standard of care. Other reconstructive options are available, although these options have drawbacks in terms of both form and function (Lake et al., 2022).

### Important dimensions of social support in VCA

#### Caregivers in VCA post-operative recovery and rehabilitation

Designated caregivers are considered a vital component of social support for VCA patients to facilitate post-operative recovery (Jowsey-Gregoire and Kumnig, 2016; Kumnig and Jowsey-Gregoire, 2016; Benedict and Magill, 2018). Postoperative VCA monitoring in the years following surgery is critical to successful patient care, and many logistical factors require united efforts on behalf of patients, their caregivers, and clinicians. While specific rehabilitation needs and requirements vary according to VCA type, lengthy rehabilitation is essential in the recovery process for most VCA recipients and may include prosthetic use, adjusting to the visible allograft, and monitoring for rejection. Moreover, further revision surgeries may be needed, particularly in facial transplantation. An established support system to facilitate the extensive logistical demands of postoperative monitoring will likely improve outcomes. The magnitude of postoperative care has prompted discussion about creating useful models for the adjunct care of VCA patients; examples include assessment of quality of life, family support, and psychiatric stability, all of which at present appear to be associated with VCA patient outcomes (Jowsey-Gregoire and Kumnig, 2016).

#### Social support and mental health in VCA

In addition to establishing the extent of familial or other caregiver support for VCA candidates, preoperative screening and intensive mental health evaluation are important elements of the assessment process for potential VCA candidates. Evaluations are particularly valuable in determining the optimal level of pre-and post-operative mental health support and follow-up (Klapheke et al., 2000; Jowsey-Gregoire and Kumnig, 2016; Kumnig and Jowsey-Gregoire, 2016). Contrary to SOT where the transplanted organ remains inside the peritoneal cavity, most VCA grafts are external. This exterior change in outward appearance such as the face and the hands can present patients with psychological implications related to their sense of self (Kumnig and Jowsey-Gregoire, 2016). Adjunct care to support patients’ mental wellness may help ease the adjustment to visible changes to the body, and adequate social support is likely to improve mental health outcomes (Kumnig and Jowsey-Gregoire, 2016; van Pilsum Rasmussen et al., 2020). Currently there is no formalized approach to optimal frequency of mental health follow up in VCA, and it remains largely program dependent. Further examination of adjunct mental health support in the context of social support and its role in VCA recovery would make a valuable contribution to the literature in VCA. While a majority of VCA patients document high levels of social support through their care (van Pilsum Rasmussen et al., 2020), this presence of social support does not necessarily preclude patients from instances of depression or anxiety that may develop during the recovery process (van Pilsum Rasmussen et al., 2020). Understanding the elements of social support that are most closely tied to outcomes during the VCA process will improve approaches to patient selection and patient care.

#### Social support and adherence to immunosuppression

Adherence to immunosuppressive medication presents another important consideration in VCA. Immunosuppressant adherence is closely tied to successful outcomes in VCA and is crucial in order to prevent major complications, including rejection and graft loss. However, these medications can cause significant side effects, and social support from caregivers has been demonstrated to help recipients maintain adherence and cope with side effects, including mood changes linked to long term immunosuppressive treatment that may make it more difficult for recipients to maintain follow up regimens (Kumnig and Jowsey-Gregoire, 2016).

### Moving toward equity in VCA

#### Important lessons from SOT: Who is harmed by social support criteria?

Berry et al. conclude that the risk of further marginalization associated with the use of social support criteria is greater for individuals of low socioeconomic status, people of color, and individuals with comorbid mental health and substance use disorders (Berry et al., 2019). These demographic groups are disproportionately affected by the implicit biases and contraindications that deny them access to lifesaving procedures (Butler and Wightman, 2021). For instance, black patients are more likely to be uninsured and less likely to be evaluated for transplant (Mohottige et al., 2021). Logistical burdens range from follow-up appointments and access to transportation, to at-home care for those unable to receive support from family. In fact, many patients report the financial stress of covering costs of transportation, medications, procedure, and aftercare outweighed their fears of the transplant itself (Mohottige et al., 2021).

The fact remains that patients of color who are underinsured and who receive less formal education undergo fewer transplants relative to their rates of organ failure, which points to deficits in the current selection process and criteria for eligibility (Reese et al., 2021). In order to address and prevent the perpetuation of these inequities, policies should account for the intersection of race and ethnicity with gender, socioeconomic status, education, and health literacy (Delaney et al., 2021). Neutral transparent evaluations, evidence-based criteria, patient-provider transparency, and revisability in guidelines are some of the factors that are essential in equitable access for transplant patients (Ladin et al., 2019a). Provider confidence and consistency with the definition of social support were found to aid the transparency of waitlist decisions and provision of greater support to the patient (Ladin et al., 2019b). Furthermore, evidence-based initiatives are imperative in establishing criteria that enhance the opportunity for transplant and eliminate the potential for both latent and overt bias within the evaluation process (Berry et al., 2019).

#### Minimizing bias and subjectivity

As evident from the existing literature, social support as a construct in SOT and VCA is inconsistently defined, and multiple dimensions are often conflated (i.e., instrumental support, informational support, emotional support). Some can be easily supplemented, others less-so. In SOT, the general consensus is that social support has a role to play in transplant evaluation, but that role should be carefully circumscribed, well-substantiated, and standardized across programs (Batra and Rubman, 2019; Berry et al., 2019; Beverley and Reischer, 2019; Fuller, 2019; Goldberg and Foster, 2019; Kelly-Hedrick and Henderson, 2019; McCauley and Fox, 2019; Parent, 2019; Priest, 2019; Sharma and Johnson, 2019; Wall, 2019). Present understanding of the role of social support in transplant outcomes relies on outdated research and highlights the dearth of current research examining the association between social support and transplant outcomes (Sharma and Johnson, 2019). There is also limited information addressing individual biases and motivations of selection committee members, as well as selection committee group dynamics and decision-making processes (McCauley and Fox, 2019). However, patient selection committees would benefit from a consistent definition of social support and understanding of the weight this definition bears in their recommendation to prevent patients from exclusion and further marginalization (Beverley and Reischer, 2019; McCauley and Fox, 2019). Some have even argued from a compensatory justice stance that patients with minimal social support may actually be more deserving of a transplant in order to have more opportunities to develop social support moving forward (Priest, 2019). While these considerations may not be immediately pressing in VCA to the same extent as in SOT, they will likely become relevant in the near future.

#### Alternative approaches to social support

Several alternatives to the present role of social support in SOT have been proposed. For example, the role of social support could be shifted away from traditional, familial models to a model in which social support is provided by the programs themselves (Parent, 2019). This may be addressed by involving social workers to help patients navigate insurance, financial stressors, and other areas of perioperative support (Goldberg and Foster, 2019). This could reduce the burden of social support being placed on the patient’s community and address the disparities across different patient populations who may otherwise be deemed lacking in social support and excluded from receiving the procedure. In addition to the potential for social support as an eligibility criterion to further exclude already marginalized groups, its contribution to gender inequity merits close scrutiny (Fuller, 2019). With the role of caregiver falling more often on women than men, current expectations for social support tend to further exacerbate the gender injustices prevalent in society (Fuller, 2019). This warrants a more equitable system, not only for patients, but for caregivers as well (Fuller, 2019).

As technology evolves, lack of informational, instrumental, and even emotional support may be at least partially mitigated by mobile or social media platforms (Kelly-Hedrick and Henderson, 2019). However, the degree of emotional support that can be provided by a program may be limited and difficult to compare to relationships that have been cultivated over many years and cannot be replaced or standardized (Wall, 2019). Preexisting social support would certainly not be denied or abandoned altogether (Batra and Rubman, 2019; Goldberg and Foster, 2019; Wall, 2019). Different types of social support will need to be examined separately to measure their independent effects on the transplantation and recovery process (Batra and Rubman, 2019).

### Strengthening construct validity to improve research design in VCA

Enhanced understanding of the role and specific mechanisms of social support during the pre-and post-transplant experience and its impact on wellbeing can help to identify opportunities to improve policies and procedures, including pre-transplant assessment, preparation for transplant surgery, support during post-transplant hospitalization, discharge planning, and short-and long-term follow up care. The heterogeneous state of the VCA literature (and the transplant literature overall) presents an opportunity for revision and integration of prior theoretical approaches and models, and an effort to more clearly define the construct and its behavioral, affective, cognitive and emotional dimensions would help to inform and prioritize future VCA research design, including the conceptualization, operationalization and measurement of meaningful outcome variables. Collaboration across VCA programs will be essential in generating and assessing the evidence needed to support adoption of a consistent definition of social support.

### Future directions for social support in VCA

In the context of clinical research with human participants, a greater degree of discretion for eligibility criteria is generally considered appropriate, as the primary goals of research are to demonstrate safety and efficacy, and therapeutic benefit is not assured. Since VCA procedures vary in their developmental trajectory, individual programs at present have a greater need for flexibility in determining VCA candidacy. However, as VCA procedures eventually shift toward clinical practice, standardizing eligibility criteria, including social support, will become increasingly germane. The field of VCA has an opportunity to incorporate patient-centered, inclusive practices from the outset. By anticipating future ethical shifts from utility toward equity, the field will better support fair access, address calls for greater transparency of the VCA patient selection process and promote a transplant system that is publicly perceived as just.

## Author contributions

LK led conception and design of the conceptual analysis, with substantial contributions from OO and ER. LK, OO, and ET drafted sections of the manuscript. All authors contributed to manuscript revision and approved the submitted version.

## Conflict of interest

The authors declare that the research was conducted in the absence of any commercial or financial relationships that could be construed as a potential conflict of interest.

## Publisher’s note

All claims expressed in this article are solely those of the authors and do not necessarily represent those of their affiliated organizations, or those of the publisher, the editors and the reviewers. Any product that may be evaluated in this article, or claim that may be made by its manufacturer, is not guaranteed or endorsed by the publisher.
